# DiaNat-DB-v2: A Molecular Database of Antidiabetic Compounds from Medicinal Plants and Functional Foods

**DOI:** 10.1021/acsomega.5c09685

**Published:** 2025-11-24

**Authors:** Nancy De Jesús-Reyes, Jimena García-Vázquez, Juan F. Avellaneda-Tamayo, David Ramírez-Palma, Mehdi D. Davari, Abraham Madariaga-Mazón, Berenice Ovalle-Magallanes, José L. Medina-Franco, Karina Martinez-Mayorga

**Affiliations:** † Institute of Chemistry, Campus Merida, 7180 National Autonomous University of Mexico, Merida-Tetis Highway, Km. 4.5, Ucu, Yucatan 97357, Mexico; ‡ DIFACQUIM Research Group, Department of Pharmacy, School of Chemistry, National Autonomous University of Mexico, Avenida Universidad 3000, Mexico City 04510, Mexico; § Department of Bioorganic Chemistry, Leibniz-Institute of Plant Biochemistry, Weinberg 3, Halle 06120, Germany; ⊥ Department of Pharmacy, School of Chemistry, National Autonomous University of Mexico, Avenida Universidad 3000, Mexico City 04510, Mexico

## Abstract

Diabetes mellitus continues to be a significant health problem worldwide, and effective strategies and resources for its prevention and treatment are needed. Here, we present DiaNat-DB-v2, a refined and expanded version of the compound database that incorporates newly identified antidiabetic compounds from medicinal plants. By incorporating food-derived compounds, DiaNat-DB-v2 bridges the gap between medicinal chemistry and functional food research, creating new opportunities for nutraceutical development, personalized dietary interventions, and drug discovery. A comprehensive analysis of structural content, diversity, chemical space coverage, and safety-related metrics revealed that the updated database exhibits minimal overlap with FDA-approved drugs and contains a large proportion of unique molecular scaffolds, underscoring its originality and complementarity. Furthermore, the database has high natural-product likeness, a low incidence of structural alerts, and moderate compliance with classical druglikeness criteria. Together, these findings emphasize the value of DiaNat-DB-v2 as a resource for both health-oriented compound discovery and nutrition-focused research. DiaNat-DB-v2 is freely accessible as Supporting Information.

## Graphical abstract

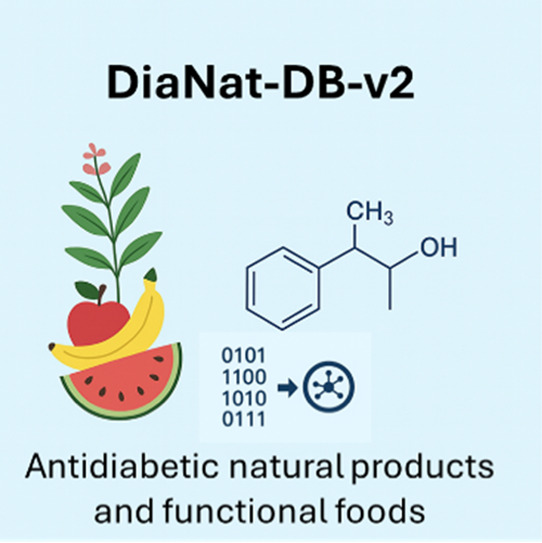

## Introduction

Diabetes mellitus (DM) is a chronic metabolic disorder marked by persistent hyperglycemia resulting from insulin deficiency, resistance, or both.^1^ The global prevalence of diabetes continues to rise, with the International Diabetes Federation (IDF) projecting 852.5 million affected adults by 2050, up from 588.7 million in 2024.^2^ This growing burden underscores the need for prevention, education and toxicologically safe alternative antidiabetic therapies with complementary mechanisms to existing drugs.

Natural products have long served as a source of bioactive molecules in general,^3^ and in diabetes mellitus in particular.^4^ Food chemicals have shown bioactive roles beyond their nutritional and organoleptic properties. For example, chemoinformatics and experimental studies have revealed that some GRAS flavor chemicals can modulate molecular targets relevant to human health.^5^ Such studies demonstrate the feasibility of applying standard cheminformatics protocols to complex food-derived data sets, supporting the rationale for expanding curated resources with compounds of nutritional and pharmacological relevance. These compounds occupy diverse and druglike regions of chemical space, with lipophilicity and structural diversity profiles comparable to approved drugs and natural products.^6^ Cheminformatics methodologies and their evolution are summarized in Gonzalez-Ponce^7^ and in the collection Milestones in Cheminformatics,^8^ offering useful guidance for newcomers to the field as well as serving as educational material. These developments have given rise to the evolving field of foodinformatics,^9^ where progress has been made, yet many research opportunities remain. The systematic characterization of the chemical space of food ingredients, similar to approaches in the pharmaceutical sciences, enables the exploration of their structural diversity and potential health benefits. Medicinal plants and functional foods hold particular promise for identifying novel antidiabetic compounds with diverse mechanisms of action.^10,11^ Numerous plant-derived metabolites, including phenolics, alkaloids, and terpenoids, have shown hypoglycemic effects via pathways such as enhancing insulin sensitivity, modulating glucose metabolism, and reducing oxidative stress.^12^ The development of a structured database for the systematic analysis of such compounds represents a timely advancement. In response to this gap, our group introduced in 2021, DiaNat-DB, a curated database of 336 antidiabetic compounds from medicinal plants, designed to facilitate chemical and pharmacological analyses.^13^ Since its launch, DiaNat-DB has supported research on the chemical space of natural antidiabetic agents. However, ongoing discoveries, advancements in computational tools, and increasing recognition of the therapeutic value of functional foods call for an updated and expanded resource.

Herein we present DiaNat-DB-v2, an expanded and enhanced version of the database that incorporates newly identified compounds from medicinal plants and food sources, with improved chemical curation and annotation. Functional foods and dietary phytochemicals with in vivo or in vitro antidiabetic activity are now included, expanding the database’s scope beyond traditional natural products and bridging medicinal chemistry with functional food research. This integration supports new directions in nutraceutical development, personalized dietary interventions, and drug discovery.^14^ Chemoinformatics methodologies were applied to curate molecular structures, annotate physicochemical properties, and analyze chemical diversity. We also present the chemical space coverage and diversity of DiaNat-DB-v2.

## Materials and Methods

### Data Collection and Literature Search

To expand the contents of DiaNat-DB with newly identified antidiabetic compounds, a systematic search was conducted in ChEMBL version 30,^15^ a publicly available bioactivity database. The keyword “diabetes” was used to retrieve relevant entries, and results were filtered with the keywords “compounds” and “natural products.” The resulting data set of compounds was classified into three categories according to their origin: synthetic compounds, natural products, and food-derived compounds.

A comprehensive literature search was performed to identify natural and food-derived compounds with antidiabetic activity. Databases queried included PubMed, SciFinder, Scopus, and ScienceDirect. Studies were included only if they provided experimental evidence of hypoglycemic, antihyperglycemic, or diabetes-complication-targeting effects. To be included in DiaNat-DB-v2, compounds needed to be reported alongside in vivo or in vitro experimental evidence of antidiabetic activity. In addition, the compound must have been evaluated as a pure compound, not as part of an extract or mixture. For in vivo studies, experiments had to be conducted in diabetic mouse models to ensure relevance to diabetes research.

### Data Curation and Annotation

For each food-derived compound identified, the following information was recorded: Simplified Molecular Input Line Entry Specification (SMILES) strings^16^ notation for molecular representation; common or IUPAC name; type of antidiabetic activity reported in the literature (diabetes-related complications, antidiabetic, antihyperglycemic, or hypoglycemic); primary food sources in which the compound is naturally found; bibliographic references from the supporting literature, using DOIs. If available, a cross-reference with FooDB (ID) was included.^17^

For natural product-derived compounds, the following data were included: SMILES string notation; common or IUPAC name; type of antidiabetic activity; plant name; botanical family; traditional use; geographic origin; and bibliographic references, using DOIs.

Best practices of data curation are paramount.^18^ Thus, chemical structures were carefully curated and verified using MarvinSketch version 22.18 ^19^ and Molecular Operating Environment (MOE), version 2024.0601,^20^ to ensure structural accuracy. For standardization RDKit toolkit version 2025.03.5^21^ and MolVS^22^ were used. Functions used included Standardizer, LargestFragmentChoser, Uncharger, and Reionizer. Compounds with valence errors and or elements other than H, B, C, N, O, F, Si, P, S, Cl, Se, Br, and I were excluded. Stereochemical information was preserved.

### Comparison with Reference Compound Databases

To contextualize the diversity and drug-likeness of DiaNat-DB-v2, it was compared with reference databases, including DrugBank, version 6.0 ^23^ for FDA-approved antidiabetic drugs, ChEMBL-reported actives against diabetic targets,^15,24^ the first version of DiaNat-DB,^13^ and a diverse subset of natural products, from the Universal Natural Products Database (UNPD,^25^ reported as UNPD-A^26^). Cross-referencing from public databases was performed using the canonical SMILES and manually verified based on the similarity results. MACCS keys and ECFP4 fingerprints were computed using the same RDKit version, parameters, and settings across all molecular data sets.

### Properties of Pharmaceutical Relevance

Six descriptors commonly used to evaluate drug-likeness^27^ were calculated: molecular weight (MW), logarithm of the partition coefficient (logP), number of hydrogen bond donors (HBD), number of hydrogen bond acceptors (HBA), topological polar surface area (TPSA), and number of rotatable bonds (RB). These properties are widely used to evaluate oral bioavailability and in the context of the empirical Rule of Five, as proposed by from Lipinski^28^ and Veber.^29^ Calculations were performed using RDKit 2025.03.5.^21^

### Constitutional Descriptors

Constitutional descriptors provide insights into the fundamental structural features. The number of heavy atoms, heteroatoms, and rings was calculated for each compound in DiaNat-DB-v2. These descriptors contribute to understanding features such as polarity, complexity, rigidity, and the presence of pharmacophoric groups.^21^ Values were calculated using RDKit 2025.03.5.^21^

### Structural Complexity

The quantification of the structural complexity is challenging and it is context-dependent.^21,22^ In this study, to evaluate the three-dimensional complexity and stereochemical features of the compounds, we calculated the fraction of sp^3^-hybridized carbon atoms (Fsp^3^) and the number of stereocenters (chiral centers). High Fsp^3^ values are empirically associated with increased clinical success rates.^23,24^ Calculations were performed using RDKit version 2025.03.5^21^ and MOE version 2024.0601.^20^

### Molecular Scaffolds

Bemis–Murcko scaffolds^30^ were extracted to analyze the core frameworks of the compounds in DiaNat-DB-v2. The Bemis–Murcko scaffold isolates the ring systems and linkers of a molecule, removing side chains to focus on the central topology. Scaffold frequencies were compared across reference data sets to identify common and unique chemotypes. Scaffold diversity was assessed by computing the scaled Shannon entropy applied to the 15 most frequent scaffolds.^31^ Extraction was performed with the RDKit toolkit version 2025.03.5.^21^

### Structural Diversity Based on Fingerprints

A set of complementary fingerprint-based molecular descriptors was computed to capture different aspects of the molecular structures: MACCS keys (166-bit dictionary-based),^32^ Morgan circular-topology-based fingerprints (1024-bit, radii 2 and 3),^33^ and MinHashed atom-pair fingerprints with chirality, up to a diameter of four bonds (MAP4Chiral).^34^ Pairwise similarities were computed within each data set with the Tanimoto coefficient as similarity index, and cumulative similarity distributions were analyzed to characterize the database-level diversity.

### Chemical Space and Chemical Multiverse Visualization

A visual representation of the chemical space based on descriptors of different nature, e.g., chemical multiverse,^35^ was generated using drug-type molecular properties and structural fingerprints. Dimensionality reduction was performed using principal component analysis (PCA)^36^ and t-distributed stochastic neighbor embedding (t-SNE),^37^ respectively. Visualizations were generated from six drug-relevant molecular descriptors and Morgan2 fingerprints, for comparison of synthetic, natural product, and food-derived antidiabetic compounds.

### Evaluation of Natural Product Likeness

The Natural Product-Likeness (NPL) score^38^ was used to evaluate how closely the compounds in DiaNat-DB-v2 resemble canonical natural product chemotypes. This machine learning-based metric estimates the probability that a molecule belongs to natural product chemical space rather than synthetic space. It has been successfully applied to natural product collections,^26,38^ food-derived compounds,^39^ synthetic libraries, and bioactive molecules.^40^

For DiaNat-DB-v2, NPL score was calculated and benchmarked against three reference sets: natural product repositories, food-related compounds, and FDA-approved drugs. This comparative framework positioned DiaNat-DB-v2 in relation to both natural product–dominated and therapeutically validated chemical spaces.

The score spans from −5 (synthetic-like) to +5 (NP-like). Rather than using fixed cutoffs, we analyzed score distributions across data sets to capture relative shifts in NP-likeness between food-derived compounds, plant-based metabolites, and synthetic comparators.

## Results and Discussion

The following section presents the main findings derived from the development and analysis of DiaNat-DB-v2. We first describe the expansion of the database and characterize its composition, with emphasis on newly incorporated food-derived molecules and their overlap with reference data sets. We then explore the chemical space covered by the database, followed by an assessment of structural diversity using molecular descriptors, scaffolds, and fingerprint-based comparisons. Finally, we evaluate the data set’s drug-likeness, and natural product-likeness. Figure 1 summarizes the overall workflow, including compound retrieval, filtering criteria, literature validation, and chemical standardization as well as the final contents of DiaNat-DB-v2.

**Figure 1:**
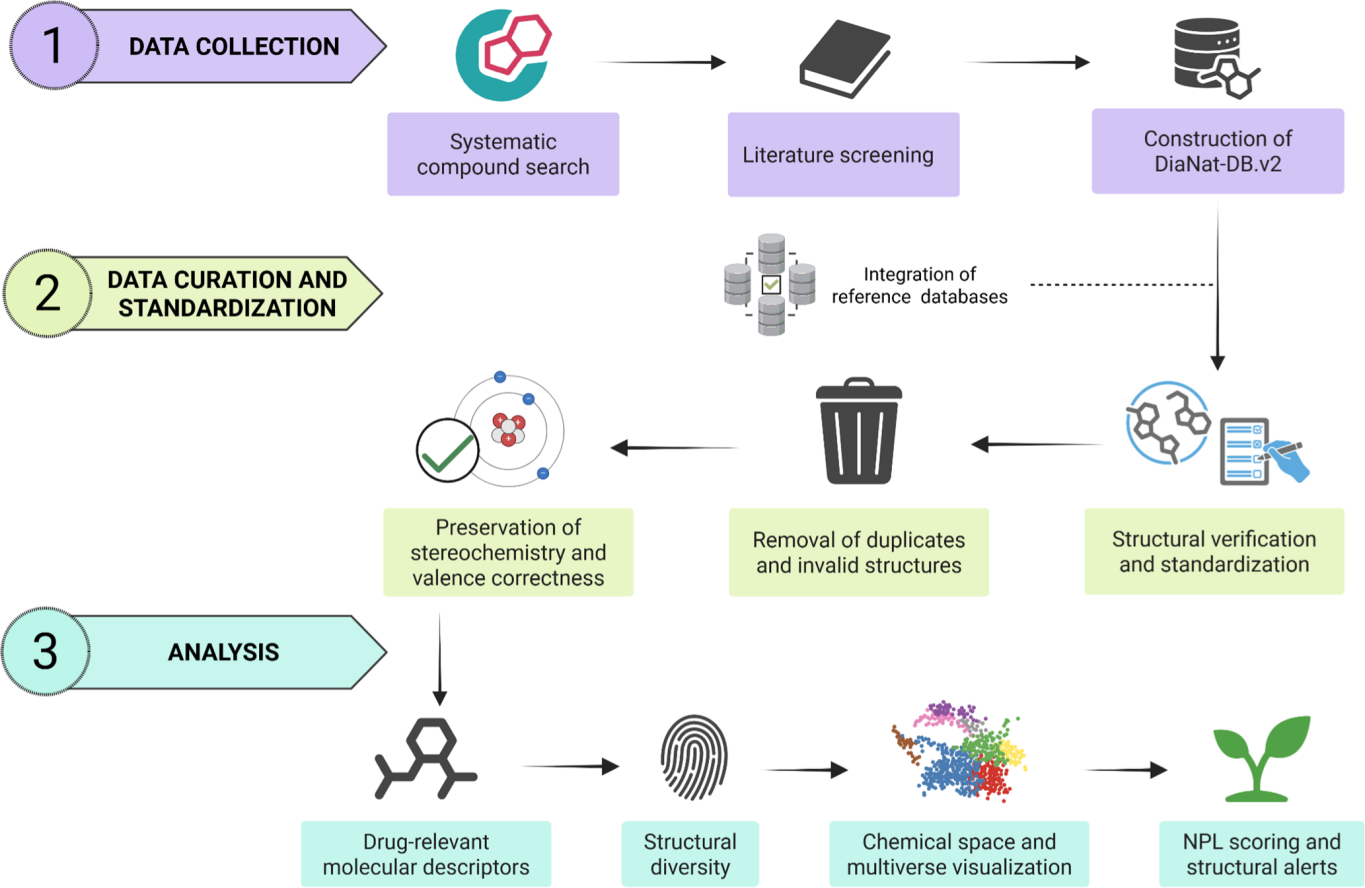
Workflow for the construction and curation of DiaNat-DB v2. Schematic overview of the database update process. Compounds were retrieved from ChEMBL using “diabetes” as a keyword and filtered by natural origin. Literature validation ensured inclusion of entries with documented in vivo or in vitro antidiabetic activity as isolated compounds. Additional metadata (e.g., source plant or food, activity type, traditional use) were manually curated, followed by chemical structure validation and descriptor calculation.

### Database Update and Composition Analysis

The updated DiaNat-DB-v2 comprises an expanded set of 654 natural product-derived compounds, of which 318 are newly added with reported antidiabetic activity, including 30 molecules obtained from food sources. Compared to the original version, which focused on plant-derived compounds, DiaNat-DB-v2 incorporates curated entries from functional foods and dietary ingredients, supported by in vivo or in vitro evidence of antidiabetic activity. This expansion not only broadens the chemical diversity of the database but also emphasizes compounds with a history of human consumption and nutritional relevance.

### Dietary Composition and Food Sources

To explore the dietary composition of DiaNat-DB-v2, we examined the food sources associated with bioactive compounds known to exhibit antidiabetic activity. Among these, milk emerged as the most frequent, followed by broccoli, citrus fruits, and red grapes. These foods are particularly rich in bioactive peptides, polyphenols, and flavonoids, molecules widely linked to the modulation of glucose metabolism, insulin signaling, and oxidative stress.^41–43^ Their prevalence highlights the growing necessity to shift to dairy- and plant-based functional foods as complementary strategies for diabetes prevention and management.^44^

In addition to analyzing individual sources, a co-occurrence network was constructed to identify food pairings that frequently appear together in the data set. The frequencies are shown as heatmap in Figure 2a and the relationships are visualized in Figure 2b. The resulting patterns revealed notable combinations, such as milk with broccoli, citrus fruits with red grapes, and eggs grouped with omega-3-rich fish, including salmon, sardines, and mackerel. These combinations guide common consumption habits, shared bioactivity profiles, and intentional pairings in nutritional interventions.

**Figure 2:**
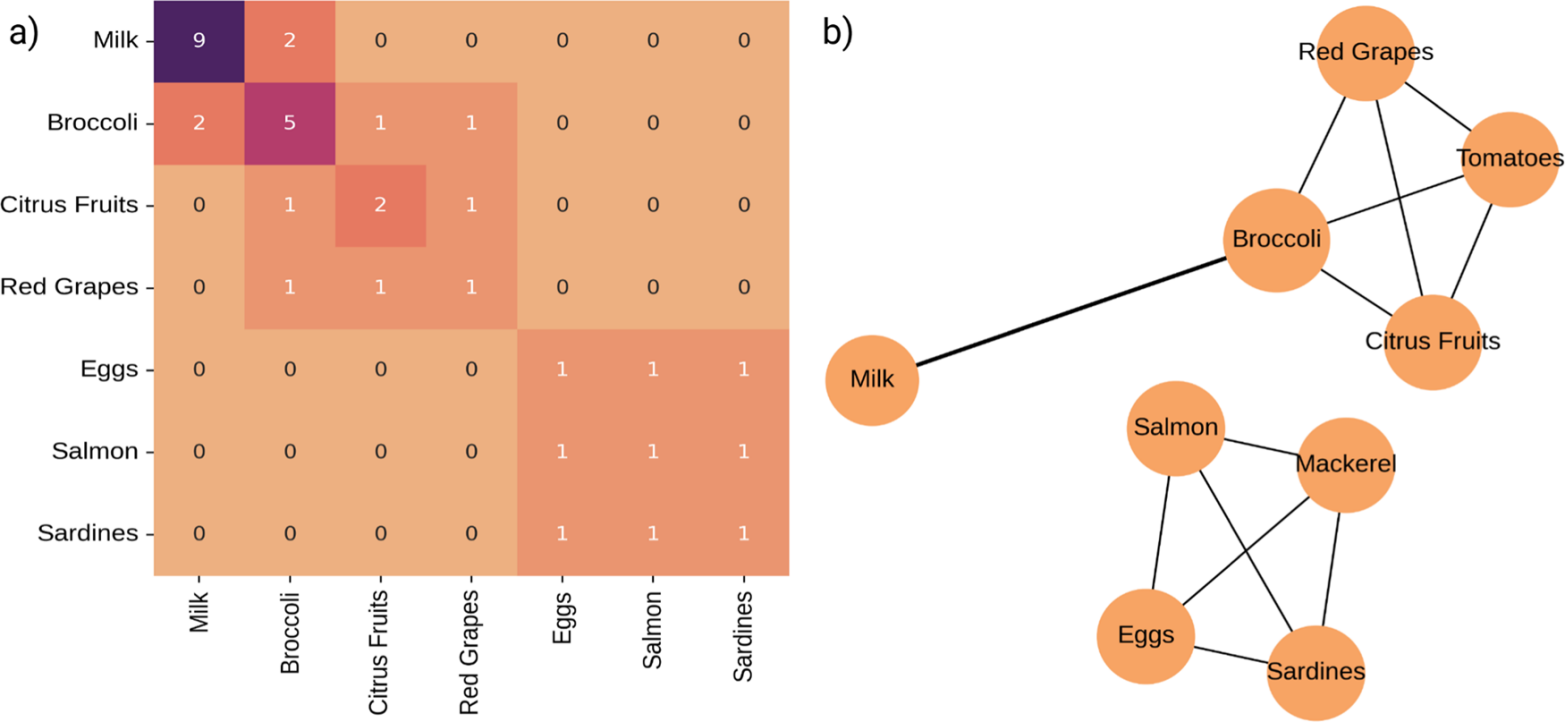
Frequency and co-occurrence of food sources associated with antidiabetic compounds in DiaNat-DB-v2. (a) Heatmaps showing the most frequently represented food sources in the database. (b) Network plot visualizes the co-occurrence of food pairs, highlighting commonly observed combinations of dietary sources across bioactive entries.

To better understand the diversity of food sources represented, the database entries were categorized by dietary groups (Figure S1). Dairy products and vegetables, particularly cruciferous varieties like broccoli and spinach, accounted for the largest proportion. Fruits rich in flavonoids also featured prominently. Protein sources, although less dominant, contributed compounds of interest such as taurine and carnosine, both of which have demonstrated insulin-sensitizing effects.^45,46^ Herbs and spices such as saffron, sage, and marjoram appear less frequently but are notable for their high polyphenol content and traditional use in metabolic regulation.^44,47^

From these observations, five functional food groupings emerged as especially relevant for antidiabetic modulation. These include dairy and cruciferous vegetables, exemplified by milk and broccoli, which are rich in peptides and dietary fiber that support glucose control.^48^ Antioxidant-rich fruits, such as citrus fruits, red grapes, tomatoes, and cherries, offer high levels of flavonoids and polyphenols.^49^ Protein- and omega-3-rich foods, such as eggs, salmon, sardines, and mackerel, contribute to both insulin sensitivity and anti-inflammatory pathways.^50^ Another group comprises polyphenol-dense foods, such as cocoa, saffron, and tarragon, known for their potent metabolic effects.^51^ Lastly, a set of herbs and spices, including sage, sweet marjoram, and tarragon, stands out for their bioactive richness and traditional medicinal use.

Beyond nutritional profiling, the inclusion of food-derived compounds in DiaNat-DB-v2 also contributes valuable structural diversity. Several entries introduce novel ring systems and acyclic motifs not commonly found in plant-based or FDA-approved data sets. This observation aligns with findings by Avellaneda-Tamayo et al.,^39^ who stated that compounds from food sources often form distinct chemical subspaces, particularly those from dairy and marine lipids (low Fsp^3^, aliphatic scaffolds) and polyphenol-rich fruits and herbs (high aromaticity, polar surface area).^52^ Many of these molecules exhibit high NPL scores and lie at the edge of conventional drug-likeness boundaries, yet remain promising due to their evolutionary adaptation to biological targets. These insights support the dual relevance of food-derived bioactive compounds in both nutritional science and medicinal chemistry, bridging dietary research with therapeutic innovation.

### Overlap with Reference Databases

A comparative analysis was performed based on curated, chiral-specific SMILES representations against reference libraries, including ChEMBL, FDA-approved drugs, and COCONUTUNPD-A. The results show that over 80% of the compounds in DiaNat-DB-v2 are unique to this data set, reinforcing its value as a complementary chemical resource. The inclusion of food-derived molecules contributes to novel chemical scaffolds and bioactivities that are largely absent from pharmaceutical databases.

### Distinct Molecular Distribution Among Natural Products

To examine the chemical diversity and uniqueness of DiaNat-DB-v2, we projected the data set into lower-dimensional spaces using PCA and t-SNE, based on molecular descriptors related to drug-likeness and topological structural fingerprints, respectively, using ECFP4. When visualized alongside reference data sets (FDA-approved drugs, ChEMBL, and UNPD-A), DiaNat-DB-v2 appeared broadly distributed, occupying peripheral regions relative to the denser regions of FDA and ChEMBL compounds. While PCA revealed partial overlap among data sets, the t-SNE plots highlighted a wider dispersion of DiaNat-DB-v2 compounds, suggesting structural heterogeneity and less clustering compared to standard pharmaceutical collections.

### DiaNat-DB-v2 Enriches the Scaffold Space of Antidiabetic Natural Products

To quantify the structural diversity of DiaNat-DB-v2, pairwise molecular similarities were calculated using two complementary fingerprinting approaches: MACCS keys (dictionary-based) and ECFP fingerprints (circular topology-based). As shown in Figure 3a, cumulative similarity distributions based on MACCS keys revealed that DiaNat-DB-v2 exhibits intermediate diversity, greater than that of FDA-approved drugs and synthetic ChEMBL compounds, but slightly lower than the natural product-rich UNPD-A data set. This pattern aligns with previous reports indicating that food-derived compounds, such as those in FooDB, often exhibit lower structural diversity due to the prevalence of lipid-dominated chemotypes.^36^ Cumulative distribution functions based on ECFP4 and ECFP6 fingerprints are shown in Supporting Information, Figure S2.

**Figure 3:**
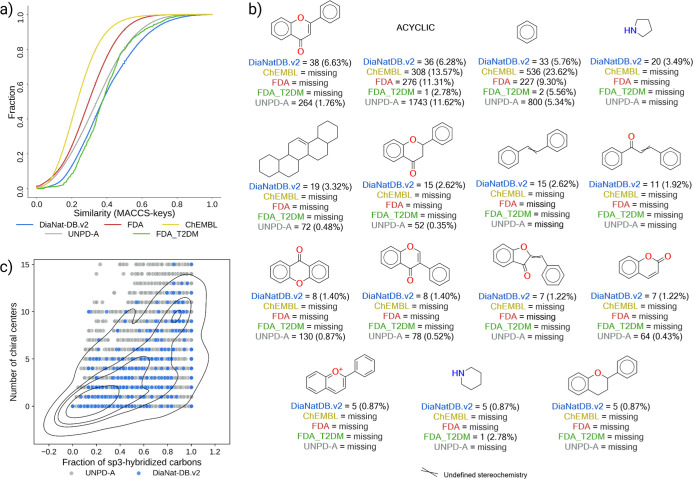
Structural diversity, scaffold profile and complexity of DiaNat-DB-v2 comparing to FDA-approved drugs, ChEMBL, and natural product reference data sets (UNPD-A, NAPROC-13). (a) Cumulative similarity distributions based on MACCS keys fingerprints. (b) The 15 most frequent Bemis–Murcko scaffolds. (c) Fraction of sp^3^-hybridized carbons (Fsp^3^) and number of chiral centers.

To explore scaffold-level novelty, a Bemis–Murcko framework analysis was conducted. The 15 most frequent scaffolds in DiaNat-DB-v2 (Figure 3b) include chromones, flavones, phenylpropanoids, and alkylated aromatics—structures frequently associated with plant-derived antidiabetic activity. Several of these scaffolds are underrepresented or absent in FDA and UNPD-A libraries, reflecting DiaNat-DB-v2’s contribution of food- and plant-specific chemotypes. Notably, 6.3% of compounds in the data set are fully acyclic, a feature also observed in FooDB and characteristic of lipidic or glycosidic natural products.

Structural features such as a higher fraction of sp^3^-hybridized carbons and increased numbers of chiral centers further support the chemical complexity of DiaNat-DB-v2. These traits are often linked to enhanced three-dimensionality, biological selectivity, and natural products–like architecture. The fraction of Fsp^3^ values and number of chiral centers across DiaNat-DB-v2 are shown in Figure 3c, illustrating the overall stereochemical richness of the data set. The corresponding plots to the other databases are shown Supporting Information, Figure S3.

Comparative analysis with NAPROC-13, a repository of pharmacologically annotated natural products,^53^ reinforces this trend: several scaffolds in DiaNat-DB-v2 were absent from standard pharmaceutical libraries, while also displaying fingerprint dissimilarity profiles consistent with scaffold novelty.

To quantify scaffold diversity, Shannon entropy analysis was applied.^31^ Results are shown in Supporting Information, Table S1. Results revealed a more even distribution of core frameworks in DiaNat-DB-v2 compared to ChEMBL and FDA-T2DM. This supports the inclusion of underexplored chemotypes—such as fused aromatic–aliphatic systems and glycosylated motifs—commonly seen in triterpenoids and natural glycosides but rarely found in synthetic drugs. Altogether, these results indicate that DiaNat-DB-v2 meaningfully expands the scaffold landscape of natural products with antidiabetic relevance, providing a valuable resource for scaffold hopping and ligand-based discovery.

The chemical space visualization is presented in Figure 4. In this projection, DiaNat-DB-v2 displayed more pronounced separation from the FDA and ChEMBL data sets, with partially exclusive clusters. This finding suggests that the structural features encoded in DiaNat-DB-v2, particularly those derived from food and plant-based compounds, are underrepresented in mainstream drug databases and contribute to a chemically complementary region of the natural product space.

**Figure 4:**
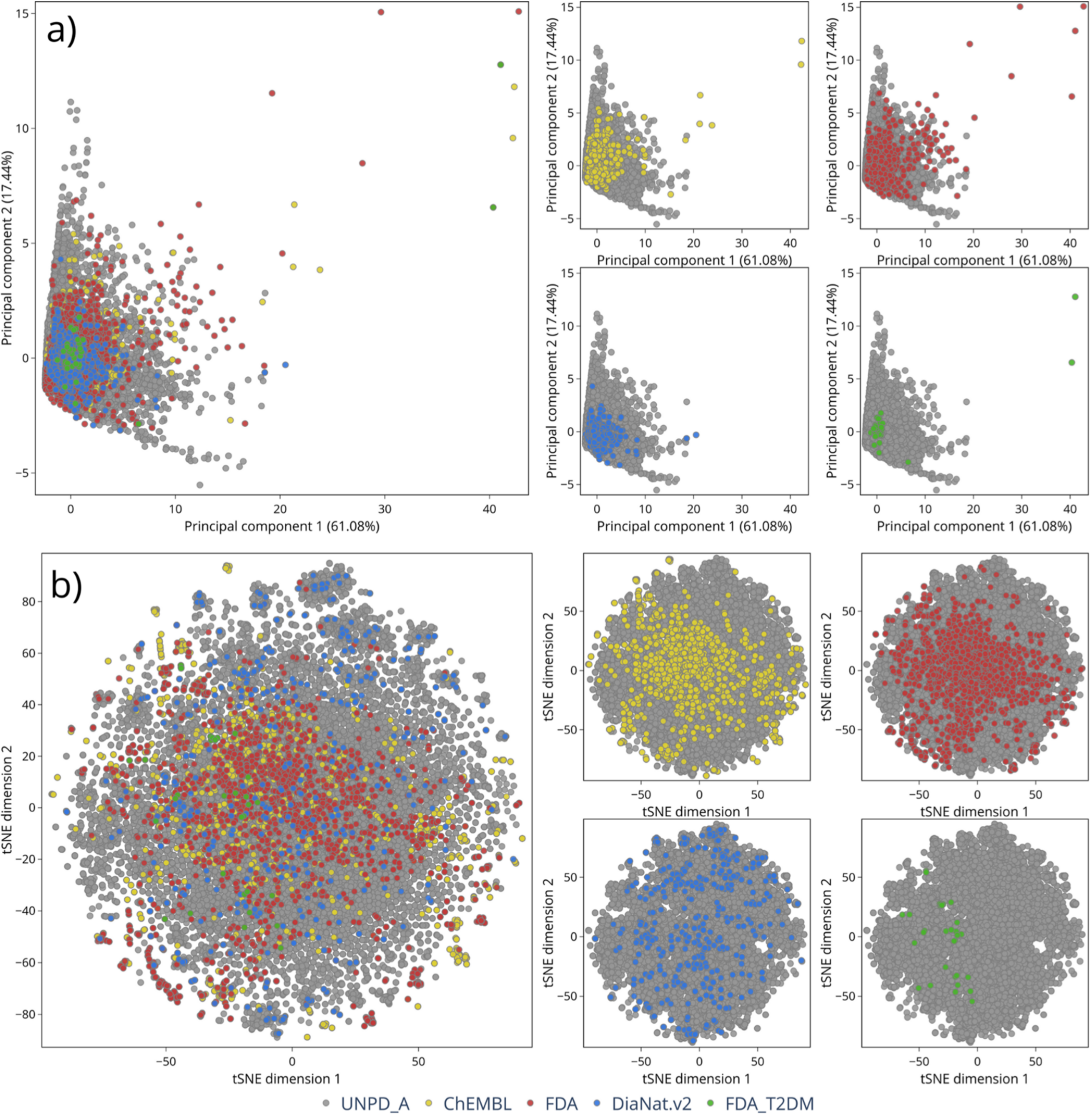
Chemical space visualizations of DiaNat-DB v2 compared to reference data sets. (a) PCA and (b) t-SNE plots based on drug-likeness-related descriptors and ECFP4 fingerprints. DiaNat-DB v2 compounds display partial overlap with natural product databases and occupy a structurally distinct region compared to FDA and ChEMBL libraries, reflecting the unique chemical landscape of plant- and food-derived molecules.

### Natural Product–Likeness

Natural Product Likeness (NPL) scores were calculated as described in the Methods section. Results are shown as Supporting Information, Figure S4. Most molecules exhibited positive NPL values, comparable to those seen in curated natural product databases such as UNPD-A and NAPROC-13. In contrast, FDA-approved drugs and ChEMBL compounds displayed broader and often lower NPL distributions. Food-derived compounds from FooDB showed intermediate values, consistent with their hybrid character. DiaNat-DB-v2, by integrating both food chemicals and medicinal plant constituents, captures a structurally meaningful middle ground between nutritional relevance and chemical richness.

### Druglikeness Profile

We evaluated the drug-likeness profile using the traditionally used Lipinski’s Rule of Five^19^ and Veber’s^20^ bioavailability guidelines. While the majority of DiaNat-DB-v2 compounds fell within traditional thresholds for molecular weight, lipophilicity, hydrogen bonding, and polar surface area, some—especially glycosides, alkaloids, and polyphenols—exceeded these limits due to their structural complexity. This pattern echoes trends observed in FooDB and NAPROC-13, reflecting the inherent nature of many plant-based and food-derived compounds.^39^ Notably, compounds such as curcumin and hesperidin, which exhibit borderline druglikeness, are nonetheless widely studied and consumed, demonstrating that traditional rules may not fully capture the relevance of naturally occurring bioactive compounds.

### Summary Profile of DiaNat-DB-v2

The overall profile of DiaNat-DB-v2, is summarized as a radar plot, shown in Figure 5a. This plot integrates chemical, structural, and safety-related metrics. The database demonstrates high compliance with NPL and a low incidence of structural alerts, alongside moderate adherence to classical druglikeness criteria. The relatively low overlap with FDA-approved drugs and a substantial proportion of unique scaffolds highlight the originality and complementary nature of the database. This multicriterion summary reinforces the relevance of DiaNat-DB-v2 for applications in both health-oriented compound discovery and nutritional research, and it is an excellent example of a database that expands the biologically relevant chemical space (BioReCS).^53^ A more detailed perspective is provided in Figure 5b, which complements the radar plot by showing the distribution of physicochemical properties across the data set. Broader ranges in molecular weight, lipophilicity, and hydrogen bonding capacity underscore the chemical diversity of DiaNat-DB-v2, emphasizing its potential for drug-likeness evaluation and for comparisons with FDA-approved drugs and other natural product data sets. The corresponding distributions for the reference databases are provided in the Supporting Information (Figure S5).

**Figure 5:**
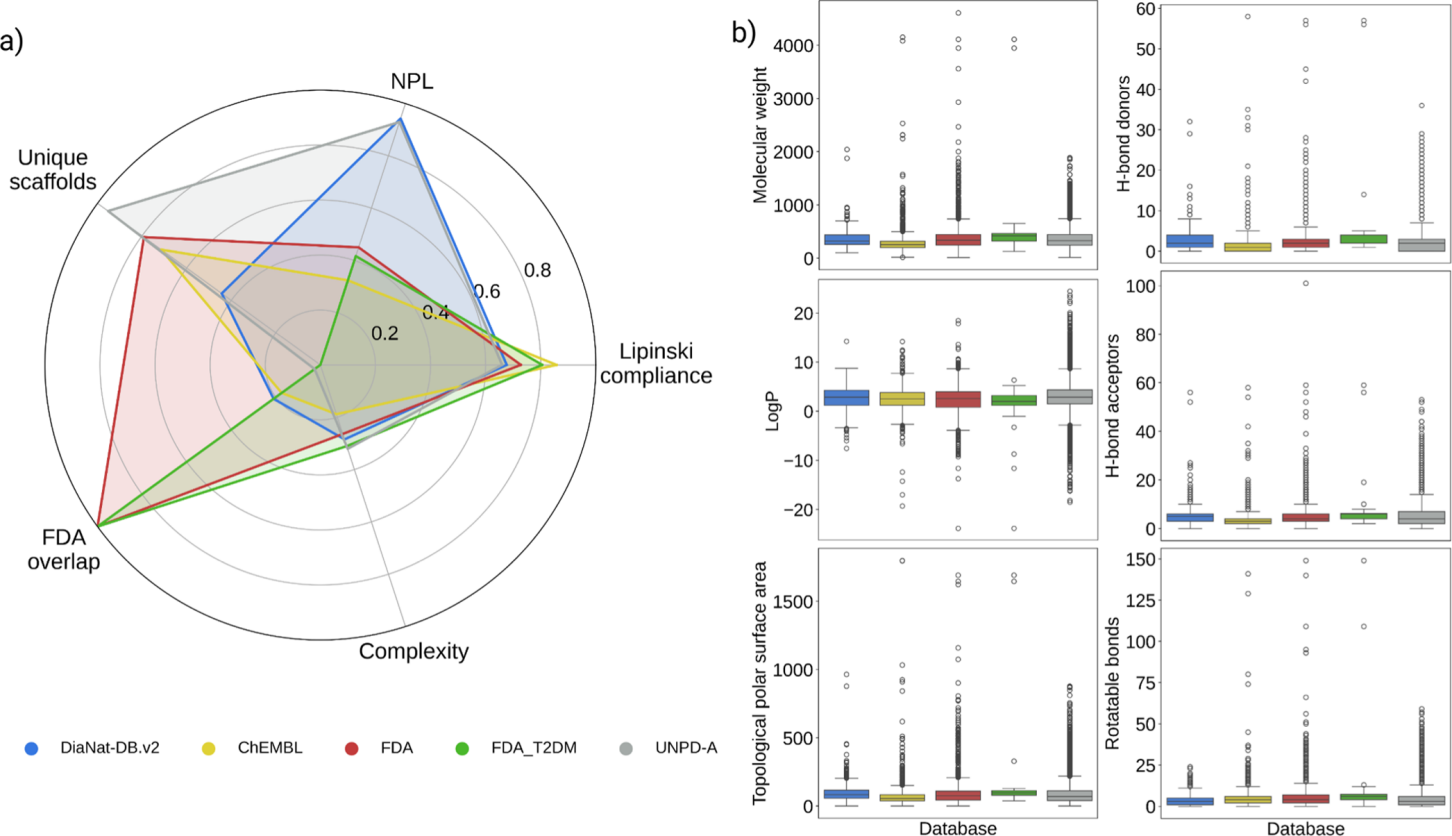
Summary profile of DiaNat-DB v2 compounds based on cheminformatic properties. (a) Radar plot integrating key metrics across the data set, including compliance with Lipinski’s and Veber’s rules, natural product-likeness scores, absence of structural alerts, scaffold uniqueness, and overlap with FDA-approved drugs. (b) Distribution plots of relevant physicochemical descriptors in DiaNat-DB v2, including molecular weight, logP, topological polar surface area (TPSA), number of hydrogen bond donors (HBD) and acceptors (HBA), and number of rotatable bonds. Together, these visualizations provide a comprehensive overview of the structural, pharmacological, and developability-related characteristics of the database.

## Conclusions and Future Directions

DiaNat-DB-v2 is an open-access molecular database comprising over 650 curated antidiabetic compounds sourced from medicinal plants and functional foods. The updated version significantly expands the original DiaNat-DB by integrating structurally diverse bioactive compounds with evidence of in vitro or in vivo hypoglycemic activity. In particular, the inclusion of food-derived compounds—especially those found in cruciferous vegetables, citrus fruits, fatty fish, and dairy—highlights the therapeutic potential of dietary sources in metabolic regulation.

Comparative analyses showed that DiaNat-DB-v2 occupies a distinct region of natural product chemical space, with a scaffold profile enriched in oxygenated fused rings, acyclic structures, and glycosylated motifs. The data set shows high NPL scores and scaffold novelty relative to FDA-approved drugs and ChEMBL compounds.

DiaNat-DB-v2 bridges pharmaceutical and nutritional domains, providing a valuable platform for health-oriented compound discovery, scaffold-based design, and functional food research. The data set supports multiple applications, including QSAR model development, virtual screening campaigns, structure–function exploration, and dietary intervention research. Future updates will incorporate new compounds, expand metadata annotations (e.g., assay conditions, microbiome interactions), and enhance the translational relevance through experimental validation of prioritized candidates. In addition, future studies will integrate tools such as QuBiLS for the calculation of molecular descriptors. This will enable large-scale diversity analyses and refined structure–activity relationship modeling.^54^

While natural products hold considerable therapeutic promise, they also carry intrinsic toxicity risks,^55^ making early safety assessment essential. Computational toxicology, together with structural alerts and predictive models, provides valuable support to experimental testing in anticipating such liabilities.^56^ These considerations will guide future efforts to ensure that compounds in DiaNat-DB-v2 are evaluated not only for efficacy but also for safety.

Together, these findings connect chemical analysis with practical dietary applications. The integration of food-derived antidiabetic compounds into DiaNat-DB-v2 not only enhances its structural and biological diversity but also supports the development of functional foods and nutraceutical strategies grounded in evidence. This alignment between nutritional science and therapeutic potential highlights the significance of food-based bioactive compounds in maintaining metabolic health.

## Supplementary Material



## Notes

Comprehensive details of the chemoinformatic characterization of DiaNat-DB-v2 in comparison with FDA-approved drugs, ChEMBL active compounds against antidiabetic targets, and the UNPD-A database, along with related data visualizations. DiaNat-DB-v2 is freely accessible as Supporting Information (PDF)
