# Supplementary material for: DiaNat-DB-v2: A Molecular Database of Antidiabetic Compounds from Medicinal Plants and Functional Foods
Source: ACS Omega. 2025 Nov 24;10(48):59782–91. doi: 10.1021/acsomega.5c09685 (PMC12771172; doi:10.1021/acsomega.5c09685)
Supplement: Supplementary file 1 [file ao5c09685_si_001.pdf]

Supporting Information

DiaNat-DB v2: A Molecular Database of Antidiabetic Compounds from Medicinal Plants and Functional Foods

Nancy De Jesús-Reyes,[1] Jimena García-Vázquez,[2] Juan F. Avellaneda-Tamayo,[2,3] David Ramírez-Palma,[1] Mehdi D. Davari,[3] Abraham Madariaga Mazón,[1] Berenice Ovalle-Magallanes,[4] José L. Medina-Franco,[2,*] and Karina Martinez-Mayorga[1,*]

[1]Institute of Chemistry, Campus Merida, National Autonomous University of Mexico, Merida-Tetis Highway, Km. 4.5, Ucu, 97357, Yucatan, Mexico

[2]DIFACQUIM Research Group, Department of Pharmacy, School of Chemistry, Universidad Nacional Autónoma de México, Avenida Universidad 3000, 04510, Mexico City, Mexico

[3]Leibniz-Institute of Plant Biochemistry, Department of Bioorganic Chemistry, Weinberg 3, 06120, Halle, Germany

[4]Department of Pharmacy, School of Chemistry, National Authonomous University of Mexico, Avenida Universidad 3000, 04510, Mexico City, Mexico

*Corresponding authors: medinajl@unam.mx (JLMF), kmtzm@unam.mx (KMM)

CONTENTS

		Page
**Figure S1**	**Food Category Distribution Bar Chart.**	S3
**Figure S2**	Cumulative distribution functions for the pairwise Tanimoto similarity using (a) ECFP4 and (b) ECFP6 as molecular representations.	S3
**Figure S3**	Fraction of sp^3-hybridized carbons (Fsp^3) and number of chiral centers for each library.	S4
**Figure S4**	Distribution of natural product likeness (NPL) score.	S5
**Figure S5**	Distribution of molecular descriptors of interest among DiaNat-DB-v2 compounds, ChEMBL antidiabetic compounds, FDA-approved drugs, and natural products in UNPD-A.	S6
**Table S1**	Shannon entropy for the 15 most common scaffolds in each database.	S8
**Table S2**	Descriptive statistics of physicochemical and constitutional descriptors computed for DiaNat-DB v2 compounds, ChEMBL synthetic compounds, FDA-approved drugs, type 2 DM FDA-approved drugs and natural products in UNPD-A..	S15

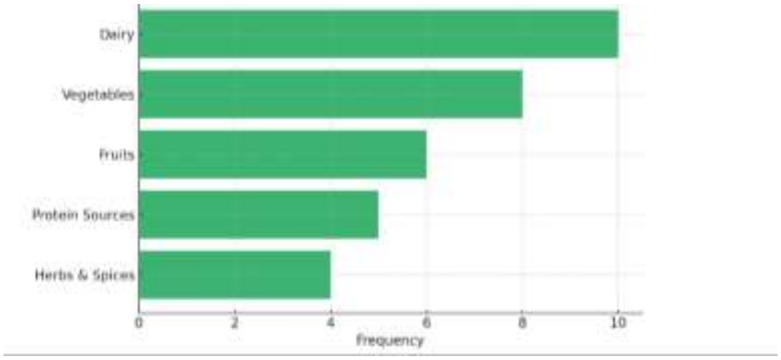

Figure S1. Food Category Distribution Bar Chart. Compares frequency of food categories (e.g., dairy, fruits, vegetables).

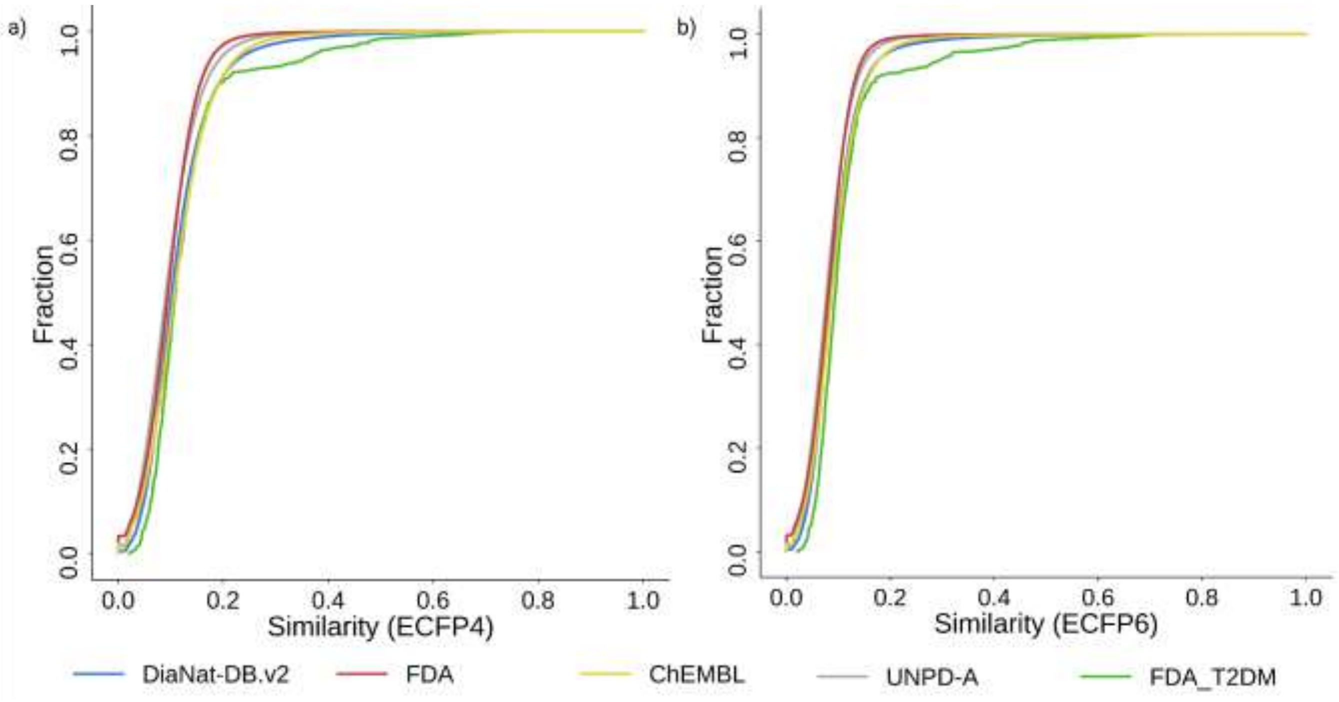

Figure S2. Cumulative distribution functions for the pairwise Tanimoto similarity using (a) ECFP4 and (b) ECFP6 as molecular representations.

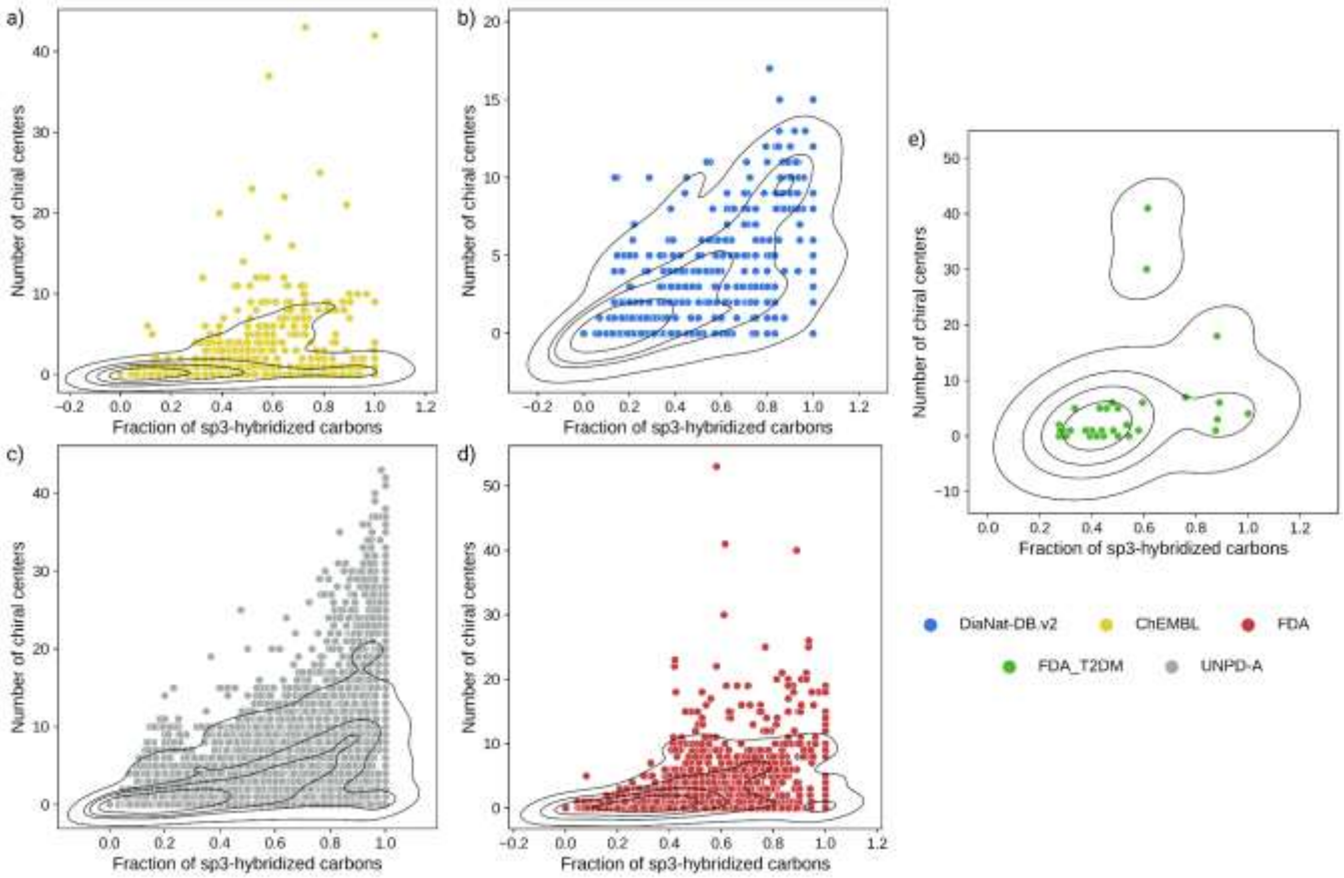

Figure S3. Fraction of sp^3-hybridized carbons (Fsp^3) and number of chiral centers for each library.

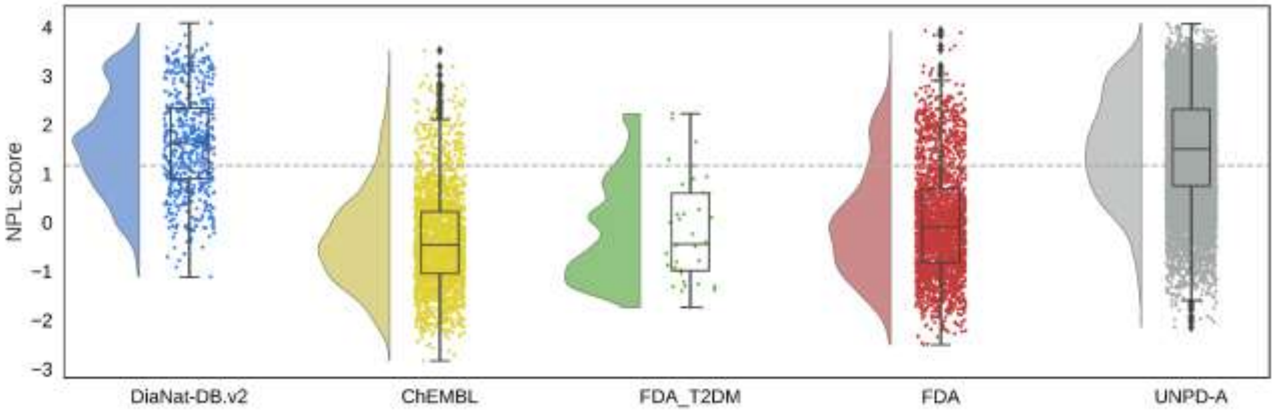

Figure S4. Distribution of natural product likeness (NPL) score. The dotted line corresponds to the mean value calculated from all datasets included in the analysis.

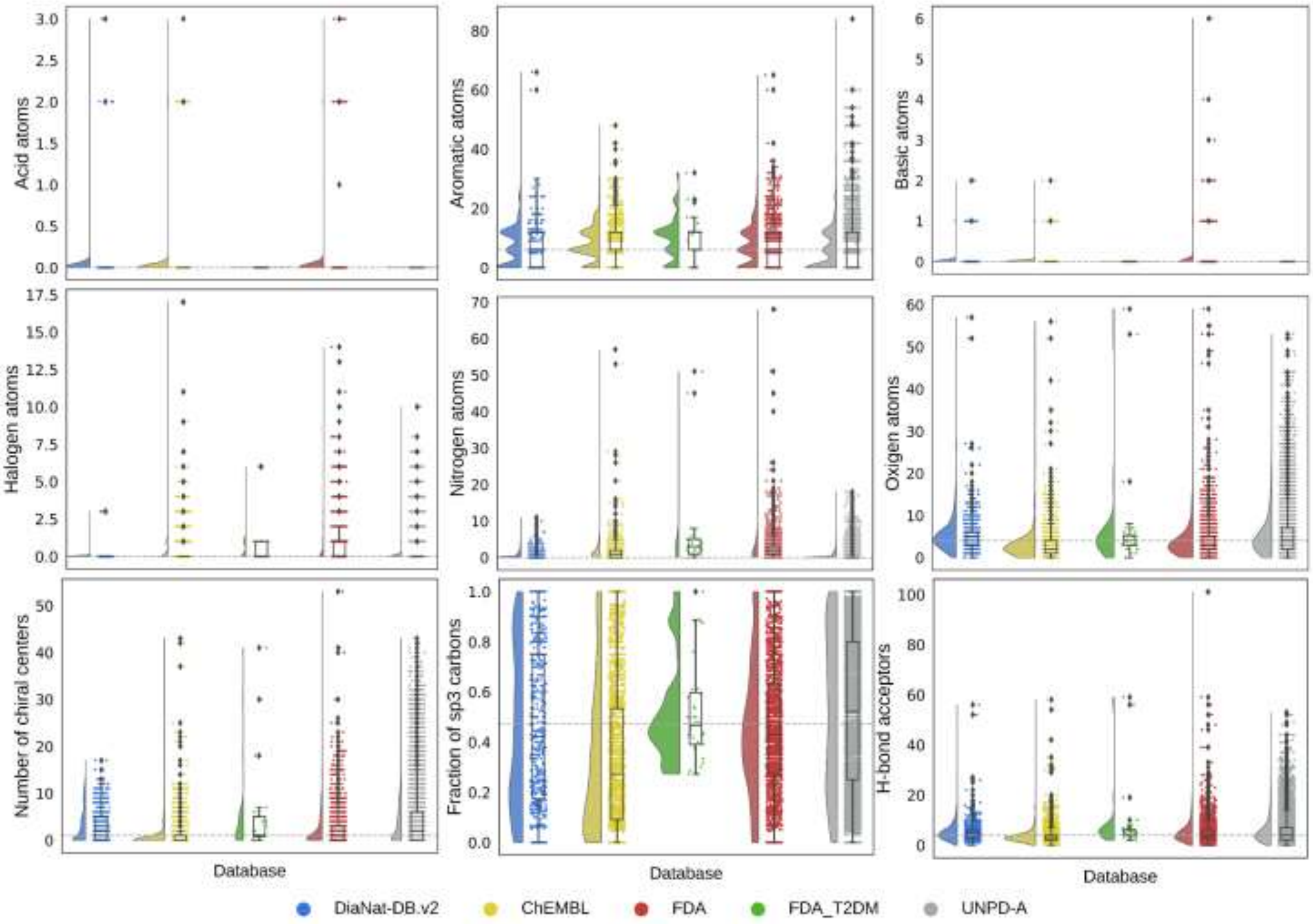
Acid atoms
Aromatic atoms
Basic atoms
Halogen atoms
Nitrogen atoms
Oxigen atoms
Number of chiral centers
Fraction of sp3 carbons
H-bond acceptors
Database
DiaNat-DB.v2
ChEMBL
FDA
FDA_T2DM
UNPD-A

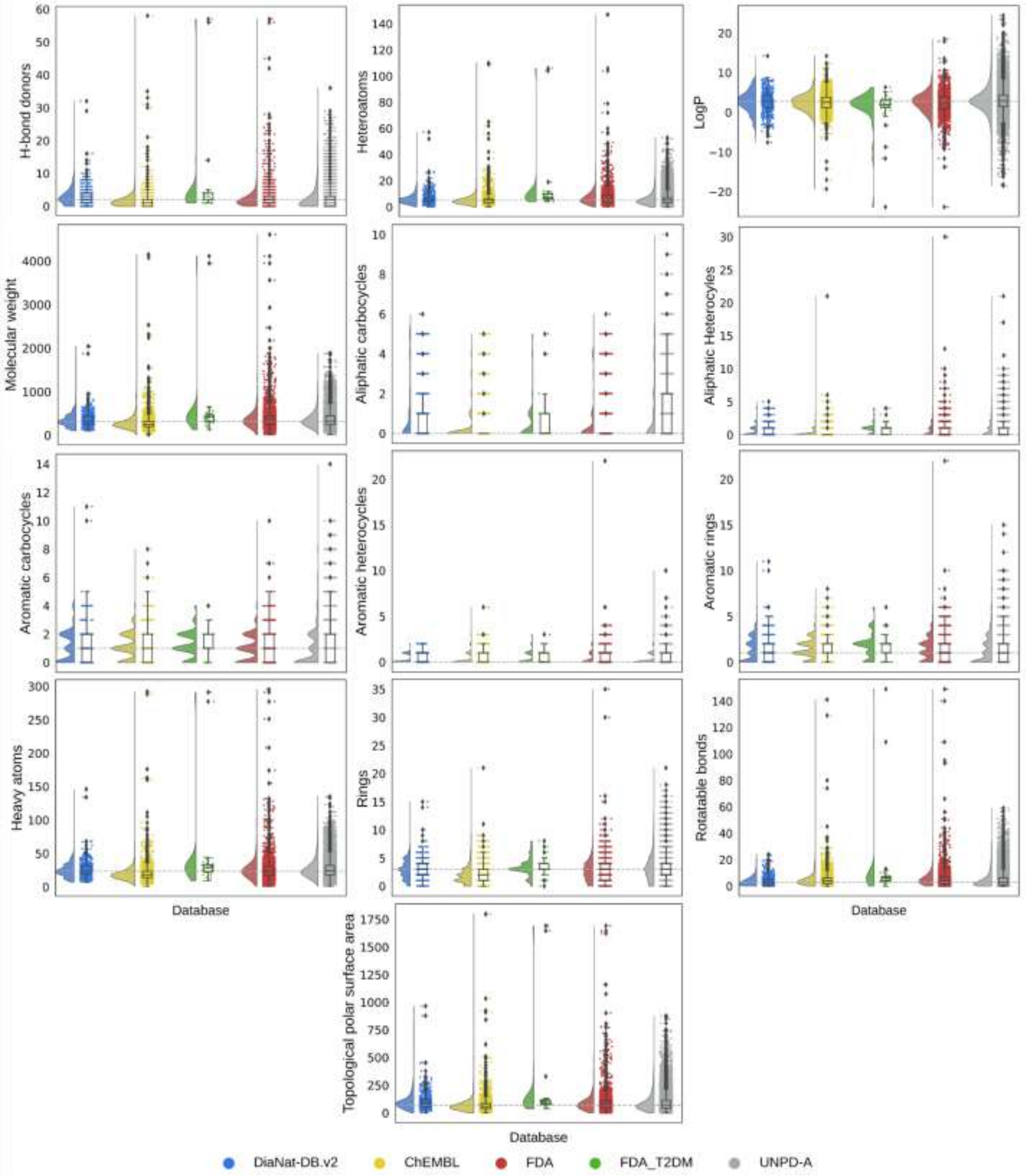

Figure S5. Distribution of molecular descriptors of interest among DiaNat-DB v2 compounds, ChEMBL antidiabetic compounds, FDA-approved drugs, and natural products in UNPD-A.

TABLES

Table S1. Descriptive statistics of physicochemical and constitutional descriptors computed for DiaNat-DB v2 compounds, ChEMBL synthetic compounds, FDA-approved drugs, type 2 DM FDA-approved drugs and natural products in UNPD-A.

Descriptor	Data set	Number of compounds	Mean	Std	Min	Q1	Median	Q3	Max
Number of acidic atoms	DiaNat-DB.v2	573	0.02	0.23	0.00	0.00	0.00	0.00	3.00
	ChEMBL	2269	0.02	0.20	0.00	0.00	0.00	0.00	3.00
	FDA	2434	0.03	0.24	0.00	0.00	0.00	0.00	3.00
	FDA-T2DM	36	0.00	0.00	0.00	0.00	0.00	0.00	0.00
	UNPD-A	14994	0.00	0.00	0.00	0.00	0.00	0.00	0.00
Number of aromatic atoms	DiaNat-DB.v2	573	7.13	7.37	0.00	0.00	6.00	12.00	66.00
	ChEMBL	2269	8.06	5.93	0.00	6.00	6.00	12.00	48.00
	FDA	2434	8.02	7.00	0.00	0.00	6.00	12.00	65.00
	FDA-T2DM	36	9.86	7.65	0.00	6.00	12.00	12.00	32.00
	UNPD-A	14994	6.19	7.43	0.00	0.00	6.00	12.00	84.00
Number of basic atoms	DiaNat-DB.v2	573	0.03	0.19	0.00	0.00	0.00	0.00	2.00
	ChEMBL	2269	0.02	0.14	0.00	0.00	0.00	0.00	2.00
	FDA	2434	0.06	0.30	0.00	0.00	0.00	0.00	6.00
	FDA-T2DM	36	0.00	0.00	0.00	0.00	0.00	0.00	0.00
	UNPD-A	14994	0.00	0.00	0.00	0.00	0.00	0.00	0.00
Number of bromine atoms	DiaNat-DB.v2	573	0.01	0.14	0.00	0.00	0.00	0.00	2.00
	ChEMBL	2269	0.03	0.19	0.00	0.00	0.00	0.00	2.00
	FDA	2434	0.02	0.17	0.00	0.00	0.00	0.00	4.00
	FDA-T2DM	36	0.03	0.17	0.00	0.00	0.00	0.00	1.00

	UNPD-A	14994	0.05	0.40	0.00	0.00	0.00	0.00	7.00
Number of chlorine atoms	DiaNat-DB.v2	573	0.01	0.07	0.00	0.00	0.00	0.00	1.00
	ChEMBL	2269	0.10	0.33	0.00	0.00	0.00	0.00	3.00
	FDA	2434	0.20	0.59	0.00	0.00	0.00	0.00	6.00
	FDA-T2DM	36	0.22	0.42	0.00	0.00	0.00	0.00	1.00
	UNPD-A	14994	0.04	0.34	0.00	0.00	0.00	0.00	10.00
Number of fluorine atoms	DiaNat-DB.v2	573	0.00	0.00	0.00	0.00	0.00	0.00	0.00
	ChEMBL	2269	0.19	0.79	0.00	0.00	0.00	0.00	17.00
	FDA	2434	0.30	1.02	0.00	0.00	0.00	0.00	14.00
	FDA-T2DM	36	0.22	1.02	0.00	0.00	0.00	0.00	6.00
	UNPD-A	14994	0.00	0.05	0.00	0.00	0.00	0.00	3.00
Number of iodine atoms	DiaNat-DB.v2	573	0.00	0.00	0.00	0.00	0.00	0.00	0.00
	ChEMBL	2269	0.01	0.12	0.00	0.00	0.00	0.00	3.00
	FDA	2434	0.05	0.40	0.00	0.00	0.00	0.00	6.00
	FDA-T2DM	36	0.00	0.00	0.00	0.00	0.00	0.00	0.00
	UNPD-A	14994	0.00	0.07	0.00	0.00	0.00	0.00	3.00
Number of nitrogen atoms	DiaNat-DB.v2	573	0.53	1.33	0.00	0.00	0.00	0.00	11.00
	ChEMBL	2269	1.67	2.57	0.00	0.00	1.00	2.00	57.00
	FDA	2434	2.52	3.39	0.00	1.00	2.00	3.00	68.00
	FDA-T2DM	36	5.08	10.78	0.00	1.00	3.00	5.00	51.00
	UNPD-A	14994	0.48	1.21	0.00	0.00	0.00	0.00	18.00
Number of	DiaNat-DB.v2	573	5.42	4.59	0.00	3.00	5.00	6.00	57.00

oxygen atoms	ChEMBL	2269	2.97	3.37	0.00	1.00	2.00	4.00	56.00
	FDA	2434	4.02	4.56	0.00	2.00	3.00	5.00	59.00
	FDA-T2DM	36	7.17	12.40	0.00	3.00	4.00	5.25	59.00
	UNPD-A	14994	5.38	5.05	0.00	2.00	4.00	7.00	53.00
Number of chiral centers	DiaNat-DB.v2	573	3.17	3.63	0.00	0.00	2.00	5.00	17.00
	ChEMBL	2269	0.92	2.66	0.00	0.00	0.00	1.00	43.00
	FDA	2434	2.31	3.93	0.00	0.00	1.00	3.00	53.00
	FDA-T2DM	36	4.56	8.45	0.00	0.75	1.00	5.00	41.00
	UNPD-A	14994	3.81	5.13	0.00	0.00	2.00	6.00	43.00
CSP3	DiaNat-DB.v2	573	0.45	0.31	0.00	0.17	0.42	0.75	1.00
	ChEMBL	2269	0.34	0.30	0.00	0.09	0.27	0.53	1.00
	FDA	2434	0.46	0.27	0.00	0.27	0.43	0.63	1.00
	FDA-T2DM	36	0.52	0.21	0.27	0.39	0.47	0.60	1.00
	UNPD-A	14994	0.52	0.31	0.00	0.25	0.52	0.80	1.00
HBA	DiaNat-DB.v2	573	5.47	4.50	0.00	3.00	5.00	6.00	56.00
	ChEMBL	2269	3.73	3.47	0.00	2.00	3.00	4.00	58.00
	FDA	2434	5.32	4.94	0.00	3.00	4.00	6.00	101.00
	FDA-T2DM	36	8.56	12.37	2.00	4.00	6.00	6.25	59.00
	UNPD-A	14994	5.58	4.95	0.00	2.00	4.00	7.00	53.00
HBD	DiaNat-DB.v2	573	3.07	2.96	0.00	1.00	2.00	4.00	32.00
	ChEMBL	2269	1.65	2.75	0.00	0.00	1.00	2.00	58.00
	FDA	2434	2.38	3.60	0.00	1.00	2.00	3.00	57.00

	FDA-T2DM	36	5.75	12.68	1.00	2.00	2.00	4.00	57.00
	UNPD-A	14994	2.51	3.17	0.00	0.00	2.00	3.00	36.00
Number of heteroatoms	DiaNat-DB.v2	573	6.00	4.65	0.00	4.00	5.00	7.00	57.00
	ChEMBL	2269	5.39	5.37	0.00	3.00	5.00	6.00	110.00
	FDA	2434	7.48	7.37	0.00	4.00	6.00	9.00	147.00
	FDA-T2DM	36	13.19	22.75	4.00	6.00	7.00	10.00	106.00
	UNPD-A	14994	6.02	5.08	0.00	3.00	5.00	7.00	53.00
LogP	DiaNat-DB.v2	573	2.65	2.72	-7.57	1.17	2.83	4.26	14.20
	ChEMBL	2269	2.49	2.26	-19.29	1.20	2.54	3.81	14.17
	FDA	2434	2.33	2.79	-23.83	0.82	2.56	3.96	18.47
	FDA-T2DM	36	0.88	5.43	-23.83	1.17	2.01	3.18	6.31
	UNPD-A	14994	2.94	3.02	-18.53	1.46	2.87	4.32	24.43
MW	DiaNat-DB.v2	573	353.42	171.92	103.06	259.08	322.07	438.17	2042.20
	ChEMBL	2269	289.15	203.71	18.01	199.95	254.07	320.25	4151.44
	FDA	2434	385.76	282.19	12.00	248.07	337.14	444.19	4606.89
	FDA-T2DM	36	598.13	851.46	129.10	321.65	420.22	466.18	4111.12
	UNPD-A	14994	371.61	196.27	16.03	246.13	330.07	445.20	1886.15
Number of halogen atoms	DiaNat-DB.v2	573	0.02	0.22	0.00	0.00	0.00	0.00	3.00
	ChEMBL	2269	0.34	0.87	0.00	0.00	0.00	0.00	17.00
	FDA	2434	0.57	1.25	0.00	0.00	0.00	1.00	14.00
	FDA-T2DM	36	0.47	1.06	0.00	0.00	0.00	1.00	6.00
	UNPD-A	14994	0.10	0.54	0.00	0.00	0.00	0.00	10.00

Number of aliphatic carbocycles	DiaNat-DB.v2	573	0.92	1.56	0.00	0.00	0.00	1.00	6.00
	ChEMBL	2269	0.22	0.70	0.00	0.00	0.00	0.00	5.00
	FDA	2434	0.52	1.14	0.00	0.00	0.00	0.00	6.00
	FDA-T2DM	36	0.56	1.11	0.00	0.00	0.00	1.00	5.00
	UNPD-A	14994	0.96	1.48	0.00	0.00	0.00	2.00	10.00
Number of aliphatic heterocycles	DiaNat-DB.v2	573	0.64	0.93	0.00	0.00	0.00	1.00	5.00
	ChEMBL	2269	0.28	0.79	0.00	0.00	0.00	0.00	21.00
	FDA	2434	0.75	1.25	0.00	0.00	0.00	1.00	30.00
	FDA-T2DM	36	1.03	1.00	0.00	0.00	1.00	1.00	4.00
	UNPD-A	14994	0.86	1.26	0.00	0.00	0.00	1.00	21.00
Number of aromatic carbocycles	DiaNat-DB.v2	573	1.13	1.24	0.00	0.00	1.00	2.00	11.00
	ChEMBL	2269	1.13	0.97	0.00	0.00	1.00	2.00	8.00
	FDA	2434	1.02	0.99	0.00	0.00	1.00	2.00	10.00
	FDA-T2DM	36	1.28	0.97	0.00	1.00	1.00	2.00	4.00
	UNPD-A	14994	0.96	1.27	0.00	0.00	0.00	2.00	14.00
Number of aromatic heterocycles	DiaNat-DB.v2	573	0.30	0.51	0.00	0.00	0.00	1.00	2.00
	ChEMBL	2269	0.34	0.64	0.00	0.00	0.00	1.00	6.00
	FDA	2434	0.50	0.88	0.00	0.00	0.00	1.00	22.00
	FDA-T2DM	36	0.50	0.74	0.00	0.00	0.00	1.00	3.00
	UNPD-A	14994	0.32	0.60	0.00	0.00	0.00	1.00	10.00
Number of aromatic rings	DiaNat-DB.v2	573	1.42	1.44	0.00	0.00	1.00	2.00	11.00
	ChEMBL	2269	1.46	1.10	0.00	1.00	1.00	2.00	8.00

	FDA	2434	1.51	1.36	0.00	0.00	1.00	2.00	22.00
	FDA-T2DM	36	1.78	1.42	0.00	1.00	2.00	2.00	6.00
	UNPD-A	14994	1.28	1.49	0.00	0.00	1.00	2.00	15.00
Number of heavy atoms	DiaNat-DB.v2	573	25.42	12.36	7.00	19.00	23.00	31.00	146.00
	ChEMBL	2269	20.04	14.39	1.00	14.00	18.00	23.00	292.00
	FDA	2434	26.59	19.46	1.00	17.00	23.00	31.00	295.00
	FDA-T2DM	36	41.81	60.12	9.00	22.75	29.00	33.00	291.00
	UNPD-A	14994	26.38	13.90	1.00	18.00	24.00	32.00	135.00
Number of rings	DiaNat-DB.v2	573	2.98	1.82	0.00	2.00	3.00	4.00	15.00
	ChEMBL	2269	1.97	1.60	0.00	1.00	2.00	3.00	21.00
	FDA	2434	2.79	2.07	0.00	1.00	3.00	4.00	35.00
	FDA-T2DM	36	3.36	1.66	0.00	3.00	3.00	4.00	8.00
	UNPD-A	14994	3.09	2.19	0.00	2.00	3.00	4.00	21.00
Number of rotable bonds	DiaNat-DB.v2	573	3.96	3.85	0.00	1.00	3.00	5.00	24.00
	ChEMBL	2269	4.99	6.40	0.00	2.00	4.00	6.00	141.00
	FDA	2434	6.00	8.00	0.00	2.00	4.00	7.00	149.00
	FDA-T2DM	36	12.67	29.14	0.00	4.00	6.00	7.25	149.00
	UNPD-A	14994	4.74	6.02	0.00	1.00	3.00	6.00	59.00
TPSA	DiaNat-DB.v2	573	98.93	79.34	0.00	57.53	83.63	116.45	964.35
	ChEMBL	2269	70.50	82.05	0.00	37.30	55.40	83.47	1798.18
	FDA	2434	94.58	107.08	0.00	43.37	74.60	109.81	1690.64
	FDA-T2DM	36	187.91	366.87	37.30	78.14	98.22	114.09	1690.64

	UNPD-A	14994	90.78	82.74	0.00	40.46	69.67	112.05	877.36

Table S5. Shannon entropy for the 15 most common scaffolds in each database

Database	Scaffold	Frequency	Pi	SE(i)	Shannon entropy
DiaNat-DB.v2	O=c1cc(-c2ccccc2)oc2ccccc12	6.632	0.164	-0.428	0.428
DiaNat-DB.v2	ACYCLIC	6.283	0.155	-0.417	0.417
DiaNat-DB.v2	c1ccccc1	5.759	0.142	-0.400	0.400
DiaNat-DB.v2	C1CCNC1	3.490	0.086	-0.305	0.305
DiaNat-DB.v2	C1=C2C3CCCC3CCC2C2CCC3CCCCC3C2C1	3.316	0.082	-0.296	0.296
DiaNat-DB.v2	O=C1CC(c2ccccc2)Oc2ccccc21	2.618	0.065	-0.255	0.255
DiaNat-DB.v2	C(=Cc1ccccc1)c1ccccc1	2.618	0.065	-0.255	0.255
DiaNat-DB.v2	O=C(C=Cc1ccccc1)c1ccccc1	1.920	0.047	-0.209	0.209
DiaNat-DB.v2	O=c1c2ccccc2oc2ccccc12	1.396	0.034	-0.168	0.168
DiaNat-DB.v2	O=c1c(-c2ccccc2)coc2ccccc12	1.396	0.034	-0.168	0.168
DiaNat-DB.v2	O=C1C(=Cc2ccccc2)Oc2ccccc21	1.222	0.030	-0.152	0.152
DiaNat-DB.v2	O=c1ccc2ccccc2o1	1.222	0.030	-0.152	0.152
DiaNat-DB.v2	c1ccc(-c2ccc3ccccc3[o+]2)cc1	0.873	0.022	-0.119	0.119
DiaNat-DB.v2	C1CCNCC1	0.873	0.022	-0.119	0.119
DiaNat-DB.v2	c1ccc(C2CCc3ccccc3O2)cc1	0.873	0.022	-0.119	0.119
ChEMBL	c1ccccc1	23.623	0.458	-0.516	0.516
ChEMBL	ACYCLIC	13.574	0.263	-0.507	0.507
ChEMBL	c1ccncc1	1.675	0.032	-0.161	0.161
ChEMBL	c1ccc2ccccc2c1	1.587	0.031	-0.155	0.155

ChEMBL	c1ccc(C(c2ccccc2)c2ccccc2)cc1	1.410	0.027	-0.142	0.142
ChEMBL	c1ccc(Nc2ccccc2)cc1	1.410	0.027	-0.142	0.142
ChEMBL	C1CCCCC1	1.278	0.025	-0.132	0.132
ChEMBL	O=C(Nc1ccccc1)c1ccccc1	1.278	0.025	-0.132	0.132
ChEMBL	c1ccc(Oc2ccccc2)cc1	1.146	0.022	-0.122	0.122
ChEMBL	c1ccc(CSc2nc3ccccc3o2)cc1	0.970	0.019	-0.108	0.108
ChEMBL	c1ncc2ncn(C3CCCO3)c2n1	0.926	0.018	-0.104	0.104
ChEMBL	c1ccc(Cc2ccccc2)cc1	0.749	0.015	-0.089	0.089
ChEMBL	c1cnccn1	0.749	0.015	-0.089	0.089
ChEMBL	O=C(OCc1ccccc1)c1ccccc1	0.617	0.012	-0.076	0.076
ChEMBL	O=C1COc2ccccc2N1	0.573	0.011	-0.072	0.072
FDA	ACYCLIC	11.311	0.399	-0.529	0.529
FDA	c1ccccc1	9.303	0.329	-0.528	0.528
FDA	O=C1C=CC2C(=C1)CCC1C3CCCC3CCC21	1.352	0.048	-0.210	0.210
FDA	O=C1C=C2CCC3C4CCCC4CCC3C2CC1	1.107	0.039	-0.183	0.183
FDA	c1ccncc1	0.738	0.026	-0.137	0.137
FDA	c1ccc(Cc2ccccc2)cc1	0.697	0.025	-0.131	0.131
FDA	C1CCCCC1	0.492	0.017	-0.102	0.102
FDA	c1ccc2c(c1)CCC1C2CCC2CCCC21	0.492	0.017	-0.102	0.102
FDA	O=C1CN=C(c2ccccc2)c2ccccc2N1	0.451	0.016	-0.095	0.095
FDA	O=C(c1ccccc1)c1ccccc1	0.410	0.014	-0.088	0.088

FDA	c1ccc2ncccc2c1	0.410	0.014	-0.088	0.088
FDA	C1=CC(c2ccccc2)C=CN1	0.410	0.014	-0.088	0.088
FDA	O=C1C2=CC3C(=O)C=CCC3CC2Cc2ccccc21	0.410	0.014	-0.088	0.088
FDA	O=C1CC(=O)NC(=O)N1	0.369	0.013	-0.082	0.082
FDA	c1ncc2ncn(C3CCCO3)c2n1	0.369	0.013	-0.082	0.082
FDA T2DM	c1ccccc1	5.556	0.118	-0.363	0.363
FDA T2DM	c1ccc(Cc2cccc(C3CCCCO3)c2)cc1	5.556	0.118	-0.363	0.363
FDA T2DM	C1=CC(NC2CCC(OC3CCCOC3)OC2)CCC1	2.778	0.059	-0.240	0.240
FDA T2DM	ACYCLIC	2.778	0.059	-0.240	0.240
FDA T2DM	C1=CC(c2ccccc2)c2cnccc2N1	2.778	0.059	-0.240	0.240
FDA T2DM	C1CCNCC1	2.778	0.059	-0.240	0.240
FDA T2DM	O=C(CC12CC3CC(CC(C3)C1)C2)N1CCC2CC21	2.778	0.059	-0.240	0.240
FDA T2DM	O=C(CCCc1ccccc1)N1CCn2cnnc2C1	2.778	0.059	-0.240	0.240
FDA T2DM	C1CSSC1	2.778	0.059	-0.240	0.240
FDA T2DM	C1OC2COC3OCOC3C2O1	2.778	0.059	-0.240	0.240
FDA T2DM	O=C(Cc1ccccc1)NCc1ccccc1N1CCCCC1	2.778	0.059	-0.240	0.240
FDA T2DM	O=C(NC1CCCCC1)NS(=O)(=O)c1ccc(CCN2C(=O)Cc3ccccc3C2=O)cc1	2.778	0.059	-0.240	0.240
FDA T2DM	O=C(NC1CCCCC1)NS(=O)(=O)c1ccc(CCNC(=O)N2CC=CC2=O)cc1	2.778	0.059	-0.240	0.240

FDA T2DM	O=C(NC1CCCCC1)NS(=O)(=O)c1ccc(CCNC(=O)c2ccccc2)cc1	2.778	0.059	-0.240	0.240
FDA T2DM	O=C(NC1CCCCC1)NS(=O)(=O)c1ccc(CCNC(=O)c2cnccn2)cc1	2.778	0.059	-0.240	0.240
UNPD-A	ACYCLIC	11.625	0.469	-0.512	0.512
UNPD-A	c1ccccc1	5.335	0.215	-0.477	0.477
UNPD-A	O=c1cc(-c2ccccc2)oc2ccccc12	1.761	0.071	-0.271	0.271
UNPD-A	O=c1c2ccccc2oc2ccccc12	0.867	0.035	-0.169	0.169
UNPD-A	C1CCOCC1	0.854	0.034	-0.167	0.167
UNPD-A	O=c1c(-c2ccccc2)coc2ccccc12	0.520	0.021	-0.117	0.117
UNPD-A	c1ccc2[nH]ccc2c1	0.520	0.021	-0.117	0.117
UNPD-A	c1ccc2ccccc2c1	0.487	0.020	-0.111	0.111
UNPD-A	C1=C2C3CCCCC3CCC2C2CCC3CCCCC3C2C1	0.480	0.019	-0.110	0.110
UNPD-A	c1ccoc1	0.454	0.018	-0.106	0.106
UNPD-A	O=c1ccc2ccccc2o1	0.427	0.017	-0.101	0.101
UNPD-A	C1CCCCC1	0.407	0.016	-0.097	0.097
UNPD-A	C1CCC2C(C1)CCC1C3CCCC3CCC21	0.393	0.016	-0.095	0.095
UNPD-A	O=C1CC(c2ccccc2)Oc2ccccc21	0.347	0.014	-0.086	0.086
UNPD-A	O=C1c2ccccc2C(=O)c2ccccc21	0.333	0.013	-0.084	0.084

Table S6. Descriptive statistics of NPL scoring.

Dataset	count	mean	std	min	Q1	Q2	Q3	max

DiaNat-DB.v2	573	1.621	1.023	-1.114	0.892	1.617	2.344	1.621
ChEMBL	2269	-0.357	0.970	-2.833	-1.047	-0.452	0.222	-0.357
FDA	2440	0.018	1.118	-2.501	-0.822	-0.100	0.688	0.018
FDA_T2DM	36	-0.179	1.057	-1.741	-0.991	-0.440	0.599	-0.179
UNPD_A	14994	1.513	1.054	-2.150	0.748	1.505	2.322	1.513
